# The Co-existence of Different Synchronization Types in Fractional-order Discrete-time Chaotic Systems with Non–identical Dimensions and Orders

**DOI:** 10.3390/e20090710

**Published:** 2018-09-14

**Authors:** Samir Bendoukha, Adel Ouannas, Xiong Wang, Amina-Aicha Khennaoui, Viet-Thanh Pham, Giuseppe Grassi, Van Van Huynh

**Affiliations:** 1 Electrical Engineering Department, College of Engineering at Yanbu, Taibah University, Medina 42353, Saudi Arabia; 2 Department of Mathematics and Computer Science, University of Larbi Tebessi, Tebessa 12002, Algeria; 3 Institute for Advanced Study, Shenzhen University, Shenzhen 518060, China; 4 Department of Mathematics and Computer Sciences, University of Larbi Ben M’hidi, Oum El Bouaghi 04000, Algeria; 5 Modeling Evolutionary Algorithms Simulation and Artificial Intelligence, Faculty of Electrical & Electronics Engineering, Ton Duc Thang University, Ho Chi Minh City, Vietnam; 6 Dipartimento Ingegneria Innovazione, Universita del Salento, 73100 Lecce, Italy

**Keywords:** fractional discrete chaos, entropy, projective synchronization, full state hybrid projective synchronization, generalized synchronization, inverse full state hybrid projective synchronization, inverse generalized synchronization

## Abstract

This paper is concerned with the co-existence of different synchronization types for fractional-order discrete-time chaotic systems with different dimensions. In particular, we show that through appropriate nonlinear control, projective synchronization (PS), full state hybrid projective synchronization (FSHPS), and generalized synchronization (GS) can be achieved simultaneously. A second nonlinear control scheme is developed whereby inverse full state hybrid projective synchronization (IFSHPS) and inverse generalized synchronization (IGS) are shown to co-exist. Numerical examples are presented to confirm the findings.

## 1. Introduction

Discrete-time chaotic systems have been the center of attention in the fields of control [1,2] and secure communications in the last few years [3,4,5,6]. This attention can be attributed to two main characteristics. First, the chaotic nature of the dynamical systems, which seems random-like but is in fact completely determined and can be predicted once the initial conditions are known. For instance, this allows for the generation of pseudo–random sequences in secret or private-key encryption. The second interesting property is their discrete nature, which allows for simple implementation and reduced computational complexity. Among the well known discrete-time chaotic systems proposed throughout the years are the Hénon map [7], the Lozi system [8], the generalized Hénon map [9] and the Baier–Klein system [10].

In recent years, researchers have picked an interest in fractional discrete-time chaotic systems. These involve fractional calculus, where the differences in the system’s dynamics are fractional. Numerous studies have been dedicated to establishing a framework for fractional discrete calculus such as [11,12,13,14]. A good summary of the subject is given in [15].

In general, chaotic systems became of interest in science and engineering in the early 1990s after synchronization was demonstrated. The earliest studies include [16,17,18,19]. Since then, various types of synchronization have been proposed in the literature including projective synchronization (PS) [20], generalized synchronization (GS) [21], full state hybrid projective synchronization (FSHPS) [22], and many more. Some modification have also been made to these synchronization types leading, for instance, to inverse generalized synchronization (IGS) [23] and inverse FSHPS (IFSHPS) [24]. With the emergence of fractional chaotic maps such as the fractional Hénon map [25] and the fractional generalized Hénon map [26], the synchronization of such maps became of interest. Very few studies can be found on the subject including [27,28,29,30,31,32].

Naturally, curiosity grew as to the possibility of multiple synchronization types being achieved simultaneously for the states of the response system. This phenomenon is commonly referred to as the co-existence of synchronization types. Many studies can be found in the literature proposing linear and nonlinear control laws that give rise to the co-existence phenomenon for continuous-time integer-order systems [33], continuous-time fractional systems [34,35,36,37,38], and discrete-time integer-order systems [39,40,41]. However, to the best of the authors’ knowledge, no such studies have been made for fractional-order discrete-time systems. This has motivated us to examine the phenomenon and develop suitable control laws for various types co-existing.

The next section of this paper describes the model for the drive and response systems and defines the necessary notation and synchronization types. Section 3 presents the control law that guarantees the co-existence of PS, FSHPS, and GS as the control laws that establish the co-existence of IFSHPS and IGS. Section 4 presents numerical examples that confirm the validity of the findings. Finally, Section 6 summarizes the work carried out in this paper.

## 2. System Model

In order to establish the co-existence of different synchronization types in fractional order discrete-time chaotic systems, we consider the generic *n*-dimensional drive and response pair of the form

(1) $$\left\{\begin{array}{c} {}^{C}\Delta_{a}^{\upsilon }x_{i}\left(t\right)=F_{i}\left(X\left(t+\alpha -1\right)\right),\\ {}^{C}\Delta_{a}^{\upsilon }y_{i}\left(t\right)=G_{i}\left(Y\left(t+\beta -1\right)\right)+u_{i}, \end{array}\;\;\;t\in N_{a+1-\upsilon }\right.$$

where $X\left(t\right)={\left(x_{1}\left(t\right),\;\ldots ,\;x_{n}\left(t\right)\right)}^{T},$ $Y\left(t\right)={\left(y_{1}\left(t\right),\;\ldots ,\;y_{n}\left(t\right)\right)}^{T}$ represent the states of the drive and response systems, respectively, $F_{i},G_{i}$ are functions from $R^{n}$ to R for 1≤i≤n, and $u_{i},1\leq i\leq n,$ denote control parameters to be identified by means of the synchronization strategy.

The notation ${}^{C}\Delta_{a}^{\upsilon }X\left(t\right)$ denotes the υ–Caputo type delta difference of a function $X\left(t\right):N_{a}\to R$ with $N_{a}=\left\{a,a+1,a+2,\ldots \right\}$ [12], which is of the form

(2) $${}^{C}\Delta_{a}^{\upsilon }X\left(t\right)=\Delta_{a}^{-(n-\upsilon )}\Delta^{n}X\left(t\right)=\frac{1}{\Gamma \left(n-\upsilon \right)}\sum_{s=a}^{t-\left(n-\upsilon \right)}{\left(t-\sigma \left(s\right)\right)}^{\left(n-\upsilon -1\right)}\Delta^{n}X\left(s\right),$$

for υ∉N is the fractional order, $t\in N_{a+n-\upsilon }$, and $n=\left[\upsilon \right]+1$. In (2), the υ–th fractional sum of $\Delta_{s}^{n}X\left(t\right)$ is defined similar to [11] as

(3) $$\Delta_{a}^{-\upsilon }X\left(t\right)=\frac{1}{\Gamma \left(\upsilon \right)}\sum_{s=a}^{t-\upsilon }{\left(t-\sigma \left(s\right)\right)}^{\left(\upsilon -1\right)}X\left(s\right),$$

with υ>0, σ(s)=s+1. The term $t^{\left(\upsilon \right)}$ denotes the falling function defined in terms of the Gamma function Γ as

(4) $$t^{\left(\upsilon \right)}=\frac{\Gamma \left(t+1\right)}{\Gamma \left(t+1-\upsilon \right)}.$$

Let us, now, define the types of synchronization with which we are interested in our study. The idea is to show that multiple types of synchronization may exist simultaneously for a pair of fractional-order discrete-time chaotic systems.

**Definition** **1.** If there exists a controller $U={\left(u_{i}\right)}_{1\leq i\leq n}$ and either constants $\gamma \in R^{*}$, a matrix *Φ*, a map $\phi :R^{n}⟶R^{n}$, a matrix *Θ*, or a map $\varphi :R^{n}⟶R^{n}$ such that

$$\begin{array}{ccc} \lim_{t\to +\infty }∥Y\left(t\right)-\gamma X\left(t\right)∥=0 & ⟹ & \mathrm{Pair}\;(1)\;\mathrm{is}\;\mathrm{projective}\;\mathrm{synchronized}\;(\mathrm{PS}).\\ \lim_{t\to +\infty }∥Y\left(t\right)-\Phi X\left(t\right)∥=0 & ⟹ & \mathrm{Pair}\;(1)\;\mathrm{is}\;\mathrm{full}\;\mathrm{state}\;\mathrm{hybrid}\;\mathrm{projective}\;\mathrm{synchronized}\;(\mathrm{FSHPS}).\\ \lim_{t\to +\infty }∥Y\left(t\right)-\phi \left(Y\left(t\right)\right)∥=0 & ⟹ & \mathrm{Pair}\;(1)\;\mathrm{is}\;\mathrm{generalized}\;\mathrm{synchronized}\;(\mathrm{GS}).\\ \lim_{t\to +\infty }∥X\left(t\right)-\Theta Y\left(t\right)∥=0 & ⟹ & \mathrm{Pair}\;(1)\;\mathrm{is}\;\mathrm{inverse}\;\mathrm{full}\;\mathrm{state}\;\mathrm{hybrid}\;\mathrm{projective}\;\mathrm{synchronized}\;(\mathrm{IFSHPS}).\\ \lim_{t\to +\infty }∥X\left(t\right)-\varphi \left(Y\left(t\right)\right)∥=0 & ⟹ & \mathrm{Pair}\;(1)\;\mathrm{is}\;\mathrm{inverse}\;\mathrm{generalized}\;\mathrm{synchronized}\;(\mathrm{IGS}). \end{array}$$

Note that in Definition 1 above, γ is a constant used to scale the master state vector. Matrices Φ and Θ represent linear transformation of the master and slave state vectors, respectively, and are usually referred to as scaling matrices. The terms *ϕ* and *φ* denote some arbitrary maps from $R^{n}$ towards $R^{n}$. In general, these are nonlinear maps that represent scaling functions. We are now ready to present the main findings of our study.

## 3. Results

### 3.1. Co-existence of PS, FSHPS and GS

Let us consider the 2-dimensional drive system and a 3-dimensional response system given, respectively, by

(5) $${}^{C}\Delta_{a}^{\upsilon }x_{i}\left(t\right)=f_{i}\left(X\left(t+\upsilon -1\right)\right),\;\;\;i=1,2,$$

and

(6) $${}^{C}\Delta_{a}^{\upsilon }y_{i}\left(t\right)=\sum_{j=1}^{3}b_{ij}y_{j}\left(t+\upsilon -1\right)+g_{i}\left(Y\left(t+\upsilon -1\right)\right)+u_{i},\;\;\;i=1,2,3,$$

where $t\in N_{a+1-\upsilon },$ 0<υ≤1, $f_{i}:R^{2}⟶R,1\leq i\leq 2,$ $\left(b_{ij}\right)\in R^{3\times 3}$ is the linear part of the drive system, $g_{i}:R^{3}⟶R,1\leq i\leq 3,$ are nonlinear functions, and $u_{i},$ i=1,2,3, are controllers to be designed. Based on Definition 1, we may define the co-existence of PS, FSHPS and GS for the coupled systems (5) and (6) as follows.

**Definition** **2.** *It is said that PS, FSHPS and GS co-exist in the synchronization of the drive system (5) and the response systems (6) if there exist a controller* $U={\left(u_{i}\right)}_{1\leq i\leq 3}$ *, a constant* $\gamma \in R^{*}$ *, a constant matrix* $\Phi ={\left(\Phi_{ij}\right)}_{1\times 2}$ *, and nonlinear map* $\phi :R^{2}⟶R$ *such that the synchronization errors*

(7) $$\left\{\begin{array}{c} e_{1}\left(t\right)=y_{1}\left(t\right)-\gamma x_{1}\left(t\right),\\ e_{2}\left(t\right)=y_{2}\left(t\right)-\Phi \times {\left(x_{1}\left(t\right),x_{2}\left(t\right)\right)}^{T},\\ e_{3}\left(t\right)=y_{3}\left(t\right)-\phi \left(x_{1}\left(t\right),x_{2}\left(t\right)\right), \end{array}\right.$$

*all satisfy the asymptotic rule*

(8) $$\lim_{t\to +\infty }∥e_{i}\left(t\right)∥=0\;\;\;for\;i=1,2,3.$$

**Remark** **1.** *From the error system (7), it is obvious that states $y_{1}$ and $x_{1}$ are projective synchronized, $y_{2}$ is full state hybrid projective synchronized with $x_{1}$ and $x_{2}$, and $y_{3}$ is generalized synchronized with $x_{1}$ and $x_{2}$.*

We also need to state the following theorems, which are necessary for the proofs to come.

**Theorem** **1** ([42])**.** *The zero equilibrium of the linear fractional-order discrete-time system*

(9) $${}^{C}\Delta_{a}^{\upsilon }e\left(t\right)=De\left(t+\upsilon -1\right),$$

*where $e\left(t\right)={\left(e_{1}\left(t\right),\ldots ,e_{n}\left(t\right)\right)}^{T},$0<υ≤1,$D\in R^{n\times n}$ and $\forall t\in N_{a+1-\upsilon },$ is asymptotically stable if*

(10) $$\lambda \in \left\{z\in C:\;\left|z\right|<{\left(2\cos\frac{\left|\arg z\right|-\pi }{2-\upsilon }\right)}^{\upsilon }\;\mathrm{and}\;\left|\arg z\right|>\frac{\upsilon \pi }{2}\right\},$$

*for all the eigenvalues λ of D.*

Next, we propose control laws that achieve the co-existence rule (7). Let us define the matrix $B={\left(b_{ij}\right)}_{3\times 3}$.

**Theorem** **2.** *PS, FSHPS and GS co-exist for the pair (5)–(6) subject to*

(11) $$\left\{\begin{array}{cc} u_{1}= & \sum_{j=1}^{3}\left(c_{1j}-b_{1j}\right)e_{j}\left(t\right)-\sum_{j=1}^{3}b_{1j}y_{j}\left(t\right)-g_{1}\left(Y\left(t+\upsilon -1\right)\right)+\gamma f_{1}\left(X\left(t+\upsilon -1\right)\right),\\ u_{2}= & \sum_{j=1}^{3}\left(c_{2j}-b_{2j}\right)e_{j}\left(t\right)-\sum_{j=1}^{3}b_{2j}y_{j}\left(t\right)-g_{2}\left(Y\left(t+\upsilon -1\right)\right)+\Phi_{1}f_{1}\left(X\left(t+\upsilon -1\right)\right)\\ & +\Phi_{1}f_{2}\left(X\left(t+\upsilon -1\right)\right),\\ u_{3}= & \sum_{j=1}^{3}\left(c_{3j}-b_{3j}\right)e_{j}\left(t\right)-\sum_{j=1}^{3}b_{3j}y_{j}\left(t\right)-g_{3}\left(Y\left(t+\upsilon -1\right)\right)+{\;}^{C}\Delta^{\beta }\phi \left(x_{1}\left(t\right),x_{2}\left(t\right)\right), \end{array}\right.$$

*where $C={\left(c_{ij}\right)}_{3\times 3}$ is a constant matrix chosen such that all the eigenvalues $\lambda_{{}_{i}}$ of B−C satisfy*

(12) $$-2^{\upsilon }<\lambda_{i}<0,\;\;\;i=1,2,3.$$

**Proof.**  The difference equations corresponding to the error system (7) are given by

(13) $$\left\{\begin{array}{c} {}^{C}\Delta_{a}^{\upsilon }e_{1}\left(t\right)={\;}^{C}\Delta_{a}^{\upsilon }y_{1}\left(t\right)-\gamma {\;}^{C}\Delta_{a}^{\upsilon }x_{1}\left(t\right),\\ {}^{C}\Delta_{a}^{\upsilon }e_{2}\left(t\right)={\;}^{C}\Delta_{a}^{\upsilon }y_{2}\left(t\right)-\Phi {\;}^{C}\Delta_{a}^{\upsilon }{\left(x_{1}\left(t\right),x_{2}\left(t\right)\right)}^{T},\\ {}^{C}\Delta_{a}^{\upsilon }e_{3}\left(t\right)={\;}^{C}\Delta_{a}^{\upsilon }y_{3}\left(t\right)-{\;}^{C}\Delta_{a}^{\upsilon }\phi \left(x_{1}\left(t\right),x_{2}\left(t\right)\right). \end{array}\right.$$

Substituting the system nonlinearities yields

(14) $$\left\{\begin{array}{cc} {}^{C}\Delta_{a}^{\upsilon }e_{1}\left(t\right)= & \sum_{j=1}^{3}b_{1j}y_{j}\left(t+\upsilon -1\right)+g_{1}\left(Y\left(t+\upsilon -1\right)\right)+u_{1}-\gamma f_{1}\left(X\left(t+\upsilon -1\right)\right),\\ {}^{C}\Delta_{a}^{\upsilon }e_{2}\left(t\right)= & \sum_{j=1}^{3}b_{2j}y_{j}\left(t+\upsilon -1\right)+g_{2}\left(Y\left(t+\upsilon -1\right)\right)+u_{2}-\Phi_{1}f_{1}\left(X\left(t+\upsilon -1\right)\right)\\ & -\Phi_{1}f_{2}\left(X\left(t+\upsilon -1\right)\right),\\ {}^{C}\Delta_{a}^{\upsilon }e_{3}\left(t\right)= & \sum_{j=1}^{3}b_{3j}y_{j}\left(t+\upsilon -1\right)+g_{3}\left(Y\left(t+\upsilon -1\right)\right)+u_{3}-{\;}^{C}\Delta_{a}^{\upsilon }\phi \left(x_{1}\left(t\right),x_{2}\left(t\right)\right). \end{array}\right.$$

Substituting the proposed control law (11) in (14) yields

(15) $$\left\{\begin{array}{c} {}^{C}\Delta_{a}^{\upsilon }e_{1}\left(t\right)=\sum_{j=1}^{3}\left(b_{1j}-c_{1j}\right)e_{j}\left(t+\upsilon -1\right),\\ {}^{C}\Delta_{a}^{\upsilon }e_{2}\left(t\right)=\sum_{j=1}^{3}\left(b_{2j}-c_{2j}\right)e_{j}\left(t+\upsilon -1\right),\\ {}^{C}\Delta_{a}^{\upsilon }e_{3}\left(t\right)=\sum_{j=1}^{3}\left(b_{3j}-c_{3j}\right)e_{j}\left(t+\upsilon -1\right). \end{array}\right.$$

In order to show that the zero solution of (16) is globally asymptotically stable, we use the linearization method as described in Theorem 1. The error system (15) can be written in the compact form

(16) $${}^{C}\Delta_{a}^{\upsilon }e\left(t\right)=\left(B-C\right)e\left(t+\upsilon -1\right).$$

where $e\left(t\right)={\left(e_{1}\left(t\right),e_{2}\left(t\right),e_{3}\left(t\right)\right)}^{T}$. According to condition (12), it is easy to see that all the eigenvalues of the matrix B−C satisfy $\left|\arg\lambda_{i}\right|=\pi >\frac{\upsilon \pi }{2}$ and $\left|\lambda_{i}\right|<{\left(2\cos\frac{\left|\arg\lambda_{i}\right|-\pi }{2-\upsilon }\right)}^{\upsilon },$ for i=1,2,3. It, then, follows immediately from Theorem 1 that the zero solution of (16) is globally asymptotically stable and consequently, systems (5) and (6) are synchronized in 3–dimensions according to Definition 2. ☐

### 3.2. Co-existence of IFSHPS and IGS

We, now, would like to achieve similar results for the inverse synchronization types listed in Definition 1. Consider the drive and response pair of the form

(17) $$\left\{\begin{array}{cc} {}^{C}\Delta_{a}^{\upsilon }x_{i}\left(t\right)=\sum_{j=1}^{2}a_{ij}x_{j}\left(t+\upsilon -1\right)+f_{i}\left(X\left(t+\upsilon -1\right)\right), & i=1,2,\\ {}^{C}\Delta_{a}^{\upsilon }y_{i}\left(t\right)=g_{i}\left(Y\left(t+\upsilon -1\right)\right)+u_{i}, & i=1,2,3, \end{array}\right.$$

where $t\in N_{a+1-\upsilon },$ $A=\left(a_{ij}\right)\in R^{2\times 2}$ and $f_{i}:R^{2}\to R,$ 1≤i≤2, are nonlinear functions, and $g_{i}:R^{3}\to R,$ 1≤i≤3. Based on Definition 1, we can state what is meant by the co-existence of IFSHPS and IGS for (17) as summarized in the following definition.

**Definition** **3.** *IFSHPS and IGS are said to co-exist in the synchronization of the pair (17) if there exist controllers $u_{i},$i=1,2,3, a constant matrix $\Theta ={\left(\Theta_{ij}\right)}_{1\times 3}$, and a map $\varphi :R^{3}⟶R$ such that the synchronization errors*

(18) $$\left\{\begin{array}{c} e_{1}\left(t\right)=x_{1}\left(t\right)-\Theta \times {\left(y_{1}\left(t\right),y_{2}\left(t\right),y_{3}\left(t\right)\right)}^{T},\\ e_{2}\left(t\right)=x_{2}\left(t\right)-\varphi \left(y_{1}\left(t\right),y_{2}\left(t\right),y_{3}\left(t\right)\right), \end{array}\right.$$

*all satisfy the asymptotic rule*

(19) $$\lim_{t\to +\infty }e_{i}\left(t\right)=0\;\;for\;i=1,2.$$

**Remark** **2.** *From the error system (18), it is apparent that $x_{1}$ is inverse full state hybrid projective synchronized with $y_{1}\left(t\right)$, $y_{2}\left(t\right)$ and $y_{3}\left(t\right)$, and that $x_{2}\left(t\right)$ is inverse generalized synchronized with $y_{1}\left(t\right)$, $y_{2}\left(t\right)$ and $y_{3}\left(t\right)$.*

Suppose that the function *φ* can be factorized in the form

(20) $$\varphi \left(y_{1}\left(t\right),y_{2}\left(t\right),y_{3}\left(t\right)\right)=\sum_{j=1}^{3}\theta_{j}y_{j}\left(t\right)+\psi \left(y_{1}\left(t\right),y_{2}\left(t\right),y_{3}\left(t\right)\right),$$

where $\theta_{j},j=1,2,3,$ are real numbers and $\psi :R^{3}\to R$ is a nonlinear function. The error dynamics (18) yield the difference equations

(21) $$\left\{\begin{array}{cc} {}^{C}\Delta_{a}^{\upsilon }e_{1}\left(t\right)= & {\;}^{C}\Delta_{a}^{\upsilon }x_{1}\left(t\right)-\Theta_{1}{\;}^{C}\Delta_{a}^{\upsilon }y_{1}\left(t\right)-\Theta_{2}{\;}^{C}\Delta_{a}^{\upsilon }y_{2}\left(t\right)\\ & -\Theta_{3}{\;}^{C}\Delta_{a}^{\upsilon }y_{3}\left(t\right),\\ {}^{C}\Delta_{a}^{\upsilon }e_{2}\left(t\right)= & {\;}^{C}\Delta_{a}^{\upsilon }x_{2}\left(t\right)-\theta_{1}{\;}^{C}\Delta_{a}^{\upsilon }y_{1}\left(t\right)-\theta_{2}{\;}^{C}\Delta_{a}^{\upsilon }y_{2}\left(t\right)\\ & -\theta_{3}{\;}^{C}\Delta_{a}^{\upsilon }y_{3}\left(t\right)-{\;}^{C}\Delta_{a}^{\upsilon }\psi \left(y_{1}\left(t\right),y_{2}\left(t\right),y_{3}\left(t\right)\right). \end{array}\right.$$

To simplify the equations, we can define

(22) $$R_{1}=\sum_{j=1}^{2}a_{1j}x_{j}\left(t\right)+f_{1}\left(X\left(t\right)\right)-\sum_{j=1}^{3}\Theta_{j}g_{i}\left(Y\left(t\right)\right),$$

and

(23) $$R_{2}=\sum_{j=1}^{2}a_{2j}x_{j}\left(t+\upsilon -1\right)+f_{2}\left(X\left(t\right)\right)-\sum_{j=1}^{3}\theta_{j}g_{i}\left(Y\left(t\right)\right)-{\;}^{C}\Delta_{a}^{\upsilon }\psi \left(y_{1}\left(t\right),y_{2}\left(t\right),y_{3}\left(t\right)\right).$$

Using (22) and (23), (21) may be written in the reduced form

(24) $$\left\{\begin{array}{c} {}^{C}\Delta_{a}^{\upsilon }e_{1}\left(t\right)=R_{1}-\sum_{j=1}^{3}\Theta_{j}u_{j},\\ {}^{C}\Delta_{a}^{\upsilon }e_{2}\left(t\right)=R_{2}-\sum_{j=1}^{3}\theta_{j}u_{j}, \end{array}\right.$$

or more compactly as

(25) $${}^{C}\Delta_{a}^{\upsilon }e\left(t\right)=R-M\times {\left(u_{1},u_{2}\right)}^{T}-{\left(\Theta_{3}u_{3},\theta_{3}u_{3}\right)}^{T},$$

where $R={\left(R_{1},R_{2}\right)}^{T}$ and

(26) $$M=\left(\begin{array}{cc} \Theta_{1} & \Theta_{2}\\ \theta_{1} & \theta_{2} \end{array}\right).$$

To establish the co-existence of IFSHPS and IGS, we assume that *M* is invertible and denote its inverse by $M^{-1}$. The control law is, then, given by

(27) $${\left(u_{1},u_{2}\right)}^{T}=M^{-1}\times \left[\left(L-A\right)e\left(t\right)+R\right]\;\mathrm{and}\;u_{3}=0,$$

where $L\in R^{2\times 2}$ is a control matrix to be determined. Substituting (27) into Equation (25), we get

(28) $${}^{C}\Delta_{a}^{\upsilon }e\left(t\right)=\left(A-L\right)e\left(t+\upsilon -1\right).$$

The following result follows in a similar manner to Theorem 2. The proof has been omitted as it can be inferred directly from that of Theorem 2.

**Theorem** **3.** *If the control matrix L is chosen such that all the eigenvalues of A−L such that $-2^{\upsilon }<\lambda_{i}<0,$i=1,2, then IFSHPS and IGS co-exist for (17) as described in (18) subject to control law (27).*

## 4. Numerical Examples

We will now put the theoretical results presented in Section 3 to the test. We consider the 2D fractional Hénon map proposed in [25] as the drive system and the 2D fractional-order generalized Hénon map [26] as the response system. The pair is described as

(29) $$\left\{\begin{array}{c} {}^{C}\Delta_{a}^{\upsilon }x_{1}\left(t\right)=x_{2}\left(t+\upsilon -1\right)-x_{1}\left(t+\upsilon -1\right)+1-a_{1}x_{1}^{2}\left(t+\upsilon -1\right),\\ {}^{C}\Delta_{a}^{\upsilon }x_{2}\left(t\right)=b_{1}x_{1}\left(t+\upsilon -1\right)-x_{2}\left(t+\upsilon -1\right), \end{array}\right.$$

and

(30) $$\left\{\begin{array}{c} {}^{C}\Delta_{a}^{\upsilon }y_{1}\left(t\right)=-y_{1}\left(t+\upsilon -1\right)-b_{2}y_{3}\left(t+\upsilon -1\right)+u_{1}\left(t+\upsilon -1\right),\\ {}^{C}\Delta_{a}^{\upsilon }y_{2}\left(t\right)=b_{2}y_{3}\left(t+\upsilon -1\right)+y_{1}\left(t+\upsilon -1\right)-y_{2}\left(t+\upsilon -1\right)+u_{2}\left(t+\upsilon -1\right),\\ {}^{C}\Delta_{a}^{\upsilon }y_{3}\left(t\right)=1+y_{2}\left(t+\upsilon -1\right)-a_{2}y_{3}^{2}\left(t+\upsilon -1\right)-y_{3}\left(t+\upsilon -1\right)+u_{3}\left(t+\upsilon -1\right). \end{array}\right.$$

The linear and nonlinear parts of the drive system (29) and the response system (30) are given by, respectively,

$$A=\left(\begin{array}{cc} -1 & 1\\ b_{1} & -1 \end{array}\right),\;f=\left(\begin{array}{c} -a_{1}x_{1}^{2}\left(t\right)+1\\ 0 \end{array}\right),$$

and

$$B=\left(\begin{array}{ccc} -1 & 0 & -b_{2}\\ 1 & -1 & b_{2}\\ 0 & 1 & -1 \end{array}\right),\;g=\left(\begin{array}{c} 0\\ 0\\ 1-a_{2}y_{3}^{2}\left(t\right) \end{array}\right).$$

**Figure 1:**
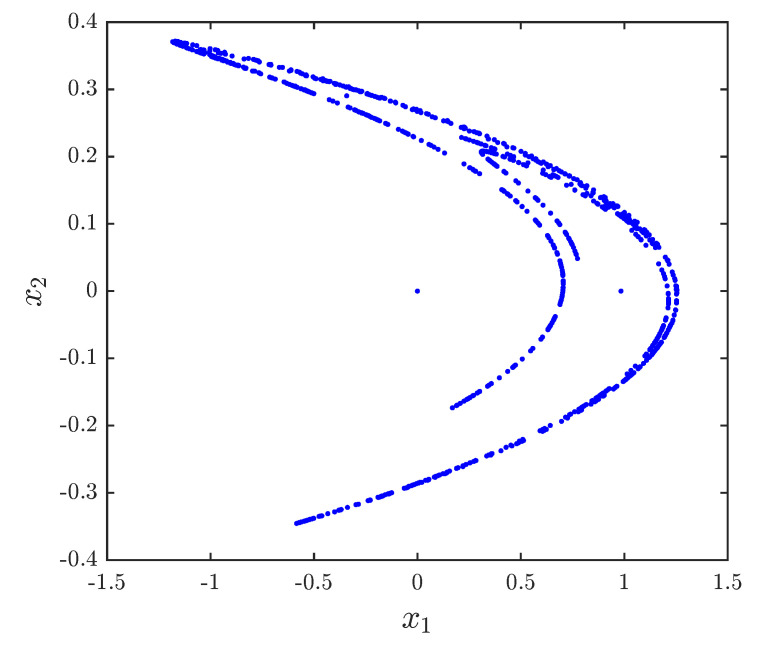
Phase space plot for the fractional Hénon map with $\left(a_{1},\;b_{1}\right)=\left(1.4,\;0.3\right)$, υ=0.984, and $\left(x_{1}\left(0\right),x_{2}\left(0\right)\right)=\left(0,0\right)$.

**Figure 2:**
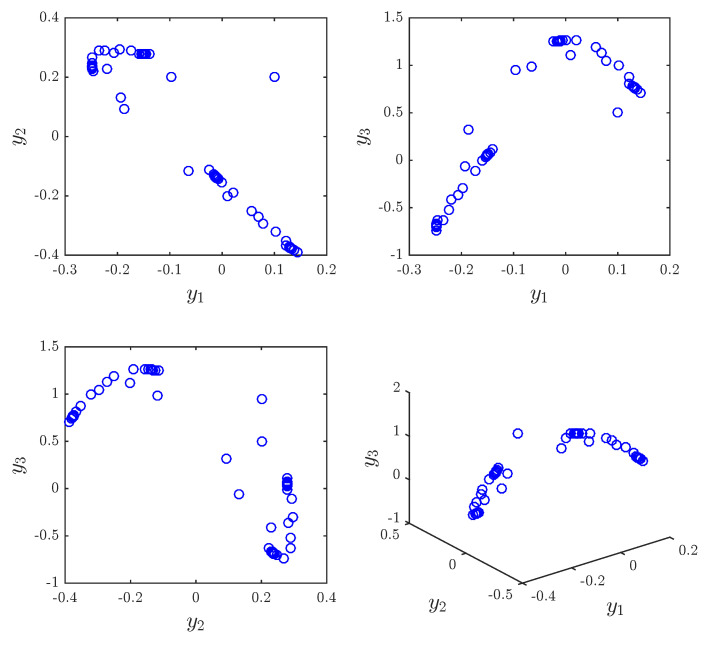
Phase portraits for the fractional generalized Hénon map with $\left(a_{2},\;b_{2}\right)=\left(0.99,\;0.2\right)$, υ=0.984, and $\left(y_{1}\left(0\right),y_{2}\left(0\right),y_{3}\left(0\right)\right)=\left(0.1,0.2,0.5\right)$.

These two systems were proposed in the literature and shown to exhibit chaotic behaviors. For instance, when $\left(a_{1},\;b_{1}\right)=\left(1.4,\;0.3\right)$, $\left(a_{2},\;b_{2}\right)=\left(0.99,\;0.2\right)$, a=0 and υ=0.984. Figure 1 and Figure 2 show the chaotic trajectories of the drive system (29) and response system (29), respectively.

Previous research in information theory has established that entropy quantifies the rate of transfer or generation of information in a particular system. In general, Kolmogorov–Sinai (KS) entropy is applied to measure dynamical systems. A direct time–series approximation of the KS entropy was proposed in [43] named ER entropy, which indicates the level of chaos in a particular system. Because calculating the exact ER entropy experimentally is difficult, an approximate entropy (ApEn) measure was introduced in [44,45]. Approximate entropy has been used to investigate chaotic systems recently [46,47].

In our work, the approximate entropy values of the drive and response systems have been calculated by using the reported scheme in [44,45]. As a brief summary of the approximation scheme, consider *N* data samples generated by our fractional map $x\left(1\right),\;x\left(2\right),\;\ldots ,\;x\left(N\right)$. The data is arranged in a sequence of vectors with an embedding dimension *m* of the form

(31) $$X\left(i\right)=\left[x\left(i\right),\;x\left(i+1\right),\;\ldots ,\;x\left(i+m-1\right)\right]\;\mathrm{with}\;1\leq i\leq N-m+1.$$

The distance between two distinct vectors $X\left(i\right)$ and $X\left(j\right)$ is denoted by $d\left(X\left(i\right),X\left(j\right)\right)$. We also define a threshold for our entropy calculation similar to [44,45] as

(32) $$r=0.2\mathrm{std}\left(x\right),$$

with std$\left(x\right)$ being the standard deviation of *x*. We, then, iterate over the regresser vectors and calculate the number of vectors *K* that yield a distance $d\left(X\left(i\right),X\left(j\right)\right)\leq r$. The approximate entropy is, then, given by

(33) $$\mathrm{ApEn}=\phi^{m}\left(r\right)-\phi^{m+1}\left(r\right),$$

where

(34) $$\phi^{m}\left(r\right)=\frac{1}{N-m-1}\sum_{i=1}^{N-m+1}\log\left(\frac{K_{i}}{N-m+1}\right).$$

The approximate entropy of the 2D fractional-order Hénon map is ApEn = 0.4159. The approximate entropy of the 2D fractional-order generalized Hénon map is ApEn = 0.0114. The results agree with trajectories illustrated in Figure 1 and Figure 2.

**Example** **1.** *The error system for the PS-FSHPS-GS synchronization scheme was described in Definition 2. We let*

(35) $$\gamma =3,\;\Phi =\left(1,3\right)\;\mathrm{and}\;\phi \left(x_{1}\left(t\right),x_{2}\left(t\right)\right)=\left(x_{1}\left(t\right)x_{2}\left(t\right)\right).$$

*Theorem 2 requires the selection of a control matrix C such that all the eigenvalues of B−C satisfy condition (12). For instance, the control matrix C can be chosen as*

(36) $$C=\left(\begin{array}{ccc} 0 & 0 & 0\\ 1 & 0 & 0\\ 0 & 1 & 0 \end{array}\right).$$

*Simply, we can show that all eigenvalues of B−C are: $\lambda_{1}=\lambda_{2}=\lambda_{3}=-1$ and therefor condition of Theorem 2 is satisfied. We can use the matrix C to construct the following controllers*

(37) $$\left\{\begin{array}{cc} u_{1}\left(t\right)= & -e_{1}\left(t\right)-b_{2}e_{3}\left(t\right)+y_{1}\left(t\right)+b_{2}y_{3}\left(t\right)+3x_{2}\left(t\right)\\ & -3x_{1}\left(t\right)+3-3a_{1}x_{1}^{2}\left(t\right),\\ u_{2}\left(t\right)= & -e_{2}\left(t\right)+b_{2}e_{3}\left(t\right)-b_{2}y_{3}\left(t\right)-y_{1}\left(t\right)+y_{2}\left(t\right)-2x_{2}\left(t\right)\\ & +\left(3b_{1}-1\right)x_{1}\left(t\right)+1-a_{1}x_{1}^{2}\left(t\right)\\ u_{3}\left(t\right)= & -e_{3}\left(t\right)-1-y_{2}\left(t\right)+a_{2}y_{3}^{2}\left(t\right)+y_{3}\left(t\right)\\ & +{\;}^{C}\Delta_{a}^{\upsilon }x_{1}\left(t\right)x_{2}\left(t\right). \end{array}\right.$$

*These controllers leads to the simplified error system*

(38) $$\left\{\begin{array}{c} {}^{C}\Delta_{a}^{\upsilon }e_{1}\left(t\right)=-e_{1}\left(t+\upsilon -1\right)-b_{2}e_{3}\left(t+\upsilon -1\right),\\ {}^{C}\Delta_{a}^{\upsilon }e_{2}\left(t\right)=-e_{2}\left(t+\upsilon -1\right)+b_{2}e_{3}\left(t+\upsilon -1\right),\\ {}^{C}\Delta_{a}^{\upsilon }e_{3}\left(t\right)=-e_{3}\left(t+\upsilon -1\right). \end{array}\right.$$

**Figure 3:**
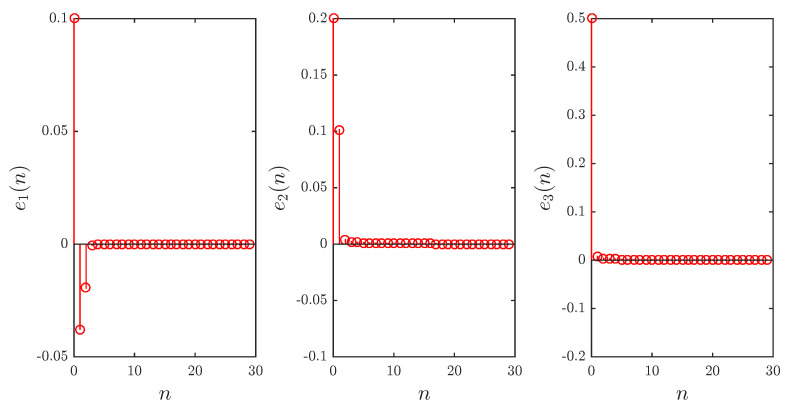
The evolution of errors over time for Example 1.

*Figure 3 shows the errors as functions of time for parameter sets $\left(a_{1},\;b_{1}\right)=\left(1.4,\;0.3\right)$ and $\left(a_{2},\;b_{2}\right)=\left(0.99,\;0.2\right)$, starting point a=0, fractional order υ=0.984, and initial errors $\left(e_{1}\left(0\right),e_{2}\left(0\right),e_{3}\left(0\right)\right)=\left(0.1,0.2,0.5\right)$. Clearly, the errors converge towards the zero solution implying that the three slave states are PS–FSHPS–GS synchronized.*

**Example** **2.** *The second case is concerned with the co-existence of IFSHPS and IGS in 2D. The error system is defined according to Definition 3 where*

(39) $$\Theta =\left(1,0,3\right)\;\mathrm{and}\;\varphi \left(y_{1}\left(t\right),y_{2}\left(t\right),y_{3}\left(t\right)\right)=y_{1}\left(t\right)+y_{2}\left(t\right)+y_{3}^{2}\left(t\right).$$

*Following the approach of Theorem 3, we start with a factorization of φ as*

(40) $$\varphi \left(y_{1}\left(t\right),y_{2}\left(t\right),y_{3}\left(t\right)\right)=\sum_{j=1}^{3}\theta_{j}y_{j}\left(t\right)+\psi \left(y_{1}\left(t\right),y_{2}\left(t\right),y_{3}\left(t\right)\right).$$

*It can be easily shown that*

(41) $$\left(\theta_{1},\theta_{2},\theta_{3}\right)=\left(1,2,0\right)\;\mathrm{and}\;\psi \left(y_{1}\left(t\right),y_{2}\left(t\right),y_{3}\left(t\right)\right)=y_{3}^{2}\left(t\right),$$

*are sufficient. The proposed synchronization scheme rearranges *Θ* and $\left(\theta_{1},\theta_{2},\theta_{3}\right)$ into the matrix*

(42) $$M=\left(\begin{array}{cc} 1 & 0\\ 1 & 2 \end{array}\right),$$

*which is invertible with inverse*

(43) $$M^{-1}=\left(\begin{array}{cc} 1 & 0\\ -\frac{1}{2} & \frac{1}{2} \end{array}\right).$$

*Theorem 3 requires the choice of a matrix L. This may be achieved with*

(44) $$L=\left(\begin{array}{cc} 1 & \frac{13}{4}\\ b_{1}-1 & -2 \end{array}\right).$$

*The controllers can, thus, be constructed according to (27) based on $R_{1}$ and $R_{2}$ defined in (22) and (23), respectively. We end up with*

(45) $$\left\{\begin{array}{cc} u_{1}\left(t\right)= & -2e_{1}-\frac{9}{4}e_{2}+x_{2}\left(t\right)-x_{1}\left(t\right)-a_{1}x_{1}^{2}\left(t\right)+y_{1}\left(t\right)\\ & +\left(b_{2}+3\right)y_{3}\left(t\right)-2-3y_{2}\left(t\right)+3a_{2}y_{3}^{2}\left(t\right),\\ u_{2}\left(t\right)= & \frac{3}{2}e_{1}+\frac{13}{8}e_{2}-y_{1}\left(t\right)+\frac{5}{2}y_{2}\left(t\right)-\left(\frac{3}{2}+b_{2}\right)y_{3}\left(t\right)\\ & -x_{2}\left(t\right)+\frac{\left(b_{1}+1\right)}{2}x_{1}\left(t\right)+\frac{1}{2}a_{1}x_{1}^{2}\left(t\right)\\ & -\frac{3a_{2}}{2}y_{3}^{2}\left(t\right)-{\frac{1}{2}}^{C}\Delta^{\beta }y_{3}^{2}\left(t\right)+1,\\ u_{3}\left(t\right)= & 0. \end{array}\right.$$

*and*

(46) $$\left\{\begin{array}{c} {}^{C}\Delta_{a}^{\upsilon }e_{1}\left(t\right)=-2e_{1}\left(t+\upsilon -1\right)-\frac{9}{4}e_{2}\left(t+\upsilon -1\right),\\ {}^{C}\Delta_{a}^{\upsilon }e_{2}\left(t\right)=e_{1}\left(t+\upsilon -1\right)+e_{2}\left(t+\upsilon -1\right). \end{array}\right.$$

**Figure 4:**
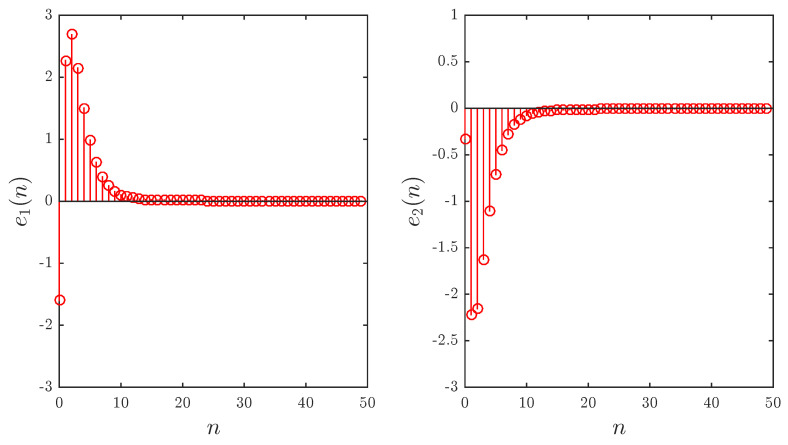
The evolution of errors over time for Example 2.

*Figure 4 depicts the stabilized states subject to parameter sets $\left(a_{1},\;b_{1}\right)=\left(1.4,\;0.3\right)$ and $\left(a_{2},\;b_{2}\right)=\left(0.99,\;0.2\right)$, starting point a=0, fractional order υ=0.984, and initial errors $\left(e_{1}\left(0\right),e_{2}\left(0\right)\right)=\left(-1.6,-0.325\right)$. It is easy to from Figure 4 that the errors converge towards zero in sufficient time proving that the controllers (45) in fact achieve IFSHPS–IGS synchronization for the pair (29).*

## 5. Discussion

In this paper, we have presented novel results concerning the co-existence of multiple synchronization types in Caputo-type fractional chaotic maps. To the best of our knowledge, the topic of co-existence has not been considered before for this type of system, which motivated this research. The synchronization types considered are rather general, which allows for multiple applications, especially in the fields of secure communications and data encryption. In fact, as we mentioned before, very few studies can be found in the literature concerning the synchronization of fractional chaotic maps, which makes this work all the more interesting.

Perhaps the most interesting studies related to the subject are [27,28,29,30,31,32]. In [27], the authors merely consider a pair of identical fractional logistic maps and propose a simple direct synchronization controller. In [28], an identical synchronization scheme is proposed based on the results of [48,49]. The authors of [29], again, consider the synchronization of identical fractional Hénon maps. The same can be said regarding [32]. As for [31], the authors propose a simple linear feedback controller suitable for a variety of maps. However, it is only shown to achieve complete synchronization, which is the most basic form of synchronization. In [30], the fractional difference operator used is different from the one used here and thus comparison is difficult.

Generally speaking, it is difficult to compare our results to those reported in the above mentioned studies as the scope of our work is much wider. In addition, we are mainly concerned with co-existence, which has not been considered before for this type of systems.

## 6. Concluding Remarks

In this work, we have shown that different types of synchronization can co-exist for fractional-order discrete-time chaotic systems. We assumed a two dimensional drive system and a three dimensional response system. The main results of the study were two fold. First, we presented a nonlinear control scheme whereby PS, FSHPS, and GS are achieved simultaneously for the three states of the response system. The stability of the zero solution, and consequently the convergence of the synchronization error, was established by means of the stability theory of linear fractional-order discrete-time systems. The second main result concerns the co-existence of IFSHPS and IGS for the same drive-response pair. The three response states are simultaneously IFSHPS synchronized with the first drive state and IGS synchronized with the second drive state. Numerical results have confirmed the findings of the study. Simulations were carried out on Matlab to ensure that the errors converge to zero subject to the proposed control laws.
